# Development of Low-Dose Disulfiram Rectal Suppository Intended for Application in Post-Treatment Lyme Disease Syndrome

**DOI:** 10.3390/pharmaceutics17070849

**Published:** 2025-06-28

**Authors:** Beáta-Mária Benkő, Bálint-Imre Szabó, Szabina Kádár, Edina Szabó, Gergő Tóth, Lajos Szente, Péter Tonka-Nagy, Romána Zelkó, István Sebe

**Affiliations:** 1 University Pharmacy Department of Pharmacy Administration, Semmelweis University, Hőgyes Endre u. 7-9, 1092 Budapest, Hungary; benko.beata@phd.semmelweis.hu; 2 Egis Pharmaceuticals Plc., R&D Directorate, 1475 Budapest, Hungary; szabo.balint.kafel3@egis.hu (B.-I.S.); tonka.nagy.peter.gabor@egis.hu (P.T.-N.); 3 Department of Applied Biotechnology and Food Science, Budapest University of Technology and Economics, Szent Gellért tér 4, 1111 Budapest, Hungary; 4 Department of Organic Chemistry and Technology, Faculty of Chemical Technology and Biotechnology, Budapest University of Technology and Economics, Műegyetem rkp. 3, 1111 Budapest, Hungary; kadar.szabina@vbk.bme.hu (S.K.); szabo.edina@vbk.bme.hu (E.S.); 5 Department of Pharmaceutical Chemistry, Semmelweis University, Hőgyes Endre u. 7-9, 1092 Budapest, Hungary; toth.gergo@semmelweis.hu; 6 Center for Pharmacology and Drug Research & Development, Semmelweis University, 1085 Budapest, Hungary; 7 CycloLab Cyclodextrin Research & Development Laboratory Ltd., Illatos út 7, 1097 Budapest, Hungary; lajos.szente@cyclolab.hu

**Keywords:** disulfiram, Lyme disease, cyclodextrin, polyethylene glycol, rectal suppository

## Abstract

**Background/Objectives**: Early diagnosis and oral or, in severe cases, intravenous antibiotics are usually effective for Lyme disease, but some patients have persistent symptoms unresponsive to standards of care, requiring alternative therapies. Disulfiram (DIS), a drug for alcoholism, is under investigation as a potential adjunctive treatment, but its low bioavailability, rapid metabolism, and safety concerns urge the development of improved formulations for clinical translation. **Methods**: Screening dissolution and permeation studies were investigated for vehicle and excipient selection, following the pharmacopeia perspectives to develop and optimize the low-dose DIS rectal suppository intended for application in post-treatment Lyme disease syndrome (PTLDS). Further characterizations were carried out by differential scanning calorimetry, X-ray diffraction, and infrared spectroscopy. **Results**: Cyclodextrin (CD) encapsulation was investigated to improve the aqueous solubility of the hydrophobic drug. The dissolution of DIS from fatty base suppository was very slow; it was remarkably improved by the molecular encapsulation of the drug with CDs. The dissolution of DIS from a water-soluble base was more favorable, but incomplete. In the polyethylene glycol (PEG) based suppositories, the addition of CDs already in a physical mixture ensured the dissolution of the drug. The presented drug delivery system relates to a novel preparation for rectal administration comprising a low-dose disulfiram with improved solubility and permeability by the PEG and hydroxypropyl-β-cyclodextrin (HPBCD) synergistic matrix. **Conclusions**: The rectal dosage form containing the drug and CD in the physical mixture is advantageous, avoiding the hepatic first-pass effect, minimizing dose-limiting toxicity, simplifying production, and fasting the availability of the repositioned drug.

## 1. Introduction

Lyme disease is a bacterial infection caused by *Borrelia burgdorferi (B. burgdorferi)*, primarily transmitted through the bites of infected ticks from the genus *Ixodes*, and is the most common vector-borne zoonotic disease; each year, there are nearly half a million cases in the USA and 65,000–85,000 in Europe [1,2,3,4]. Timely diagnosis and intervention are essential for achieving full recovery; early-stage Lyme disease is treated with a regimen of oral antibiotics such as doxycycline, amoxicillin, or cefuroxime axetil [1]. In more severe instances, especially those with neurological involvement, intravenous antibiotics may be necessary [1]. However, the treatment of Lyme disease presents several challenges, particularly regarding early diagnosis, effective antibiotic therapy, and the management of lingering symptoms after treatment [1,2]. A notable concern arises when patients continue to experience persistent symptoms, a condition referred to as Post-Treatment Lyme Disease Syndrome (PTLDS), developing in 2–40% of individuals treated for Lyme disease [1,2]. The pathophysiology of PTLDS is inadequately understood and lacks approved treatment options, and the diagnosis of this disease entity is controversial in the medical community; symptoms including chronic fatigue, widespread musculoskeletal pain, and cognitive and sleep difficulties that last for 6 months or longer following the conclusion of antibiotic therapy [1,2,5]. The behavior and life cycle of *B. burgdorferi* is not entirely understood, particularly its interactions with the immune system and its ability to persist in the body even after antibiotic treatment [1]. Thus, there is an urgent need for comprehensive strategies that address both the immediate and long-term aspects of Lyme disease to enhance a patient’s outcomes and overall quality of life [2]. Ongoing research aims to deepen the understanding of PTLDS and to identify effective therapies [2]. New clinical trials are investigating various treatment alternatives beyond conventional antibiotics, including vaccination, stem cell therapy, immunotherapy and repurposing available drugs [1,2].

The aldehyde dehydrogenase (ALDH) inhibitor, disulfiram (DIS), originally used to treat alcohol abuse, was identified in 2016 as a potent inhibitor of stationary phase persisters of *B. burgdorferi* [6,7]. Since then, considerable clinical interest (NCT03891667) [7,8,9,10,11,12] promising preclinical results [2,7,13,14,15,16] and off-label use of DIS [8,17] have demonstrated beneficial effects in the treatment of Lyme disease, especially in PTLDS [8,13]. However, the mechanism of action of DIS in treating Lyme disease is still under research; several hypotheses have been proposed to explain its potential therapeutic effects [1]. The overall biological interactions of the symmetric molecule of DIS are attributed to its sulfur content, from which free thiol groups can be formed during its decomposition, involved in thiocarbamate–thiol type reactions with free thiol groups of proteins and enzymes; or it can form chelate complexes with metal ions, strongly influencing intracellular trace element-dependent processes [18,19,20,21]. *B. burgdorferi* contains a variety of intracellular cofactors (e.g., coenzyme A reductase), metabolites (e.g., glutathione), and enzymes (e.g., thioredoxin) with thiophilic residues that can undergo modification by DIS through thiol–disulfide exchanges, affecting the flexibility and function of microbial surface membranes, thereby eliciting antimicrobial effects [13]. Similarly, *B. burgdorferi* requires Zn^2+^ and Mn^2+^ as cofactors for key biological functions, and due to the drug’s high affinity for metal ions, may inhibit microbial metabolism [13]. DIS has excellent borreliacidal activity against both the log and stationary phase *B. burgdorferi* in vitro and in vivo [13]. The minimal inhibitory concentration of DIS against the bacteria varies between 0.18 μg/mL–2.97 μg/mL [6,7,13,14]. In C3H/HeN mice, DIS restricts the further growth and dissemination of *B. burgdorferi*, like doxycycline, but DIS treatment also reduced the inflammatory microenvironment and the disease severity in the heart [13,22]. One explanation for DIS’ apparent potency compared to conventional antibiotics in the treatment of Lyme disease is its and its metabolites’ higher diffusion into biofilm [9,15,23,24,25]. The existence of biofilm is known to increase antibiotic resistance of resident microbes, which may explain relapses in previously antibiotic-treated patients [9]. Moreover, *B. burgdorferi* develops in tendons, fascia, and nerve tissues; cystic forms are unreachable for antibiotics and immunological response and can revive to active forms, and this virulence depends on the number of plasmids, because they may encode virulent DNA [12]. Thus, medication therapies have to be effective extra and intracellular, in cystic forms and round bodies, and in reaching the central nervous system and cerebrospinal fluid [12]. DIS presents the advantage of being able to penetrate all tissues, but also the cytoplasm, nucleus, and mitochondria, and passes the blood–brain barrier [12]. However, the metabolism of DIS is variable, seen in elimination kinetic studies of alcohol-dependent patients, which results from the lipid solubility of the drug, differences in plasma protein binding, and the effect of enterohepatic cycling, thus individualized DIS dosing strategies and the development of value-added drug delivery system are needed [9]. Aside from the positive preclinical results, the negative in vitro [14] and in vivo outcomes are worth mentioning; giving DIS to mice in oral gavage in antibiotic combination therapies was not effective at eradicating *B. burgdorferi*; the persistent infection rates were similar to those of control mice [16]. Another issue in the concentration–response profile of DIS was observed, as at low concentrations (ranging from 0.625 to 10 μM) the profile was sigmoidal, and at higher concentrations (ranging from 25 to 100 μM), the drug lost its efficacy and exhibited a U- or bell-shaped curve; the controversial effects are explained by the colloidal forms of DIS at high concentrations [13,26]. The pharmacokinetics (PK) and pharmacodynamics of a daily 500 mg dose of oral DIS explain the success in alcohol dependence but fail to achieve the same safety and efficacy profile in the new potential indication scope [17,19,26]. Patients experienced toxic effects or side effects, mainly concerning the nervous system (neuropathies, headaches, dizziness, difficulty of concentration and expression, sleep disturbance, general pain increase, increase in general fatigue), which could be due to high DIS doses, similar to those used for alcohol-dependent patients, or to Herxheimer reactions triggered by the DIS-induced death of *B. burgdorferi* [8,11,17,27,28,29]. The Jarisch–Herxheimer reaction is an acute, transient inflammatory response that may occur after the initiation of antibiotic therapy for Lyme disease, attributed to the release of endotoxins from dying spirochetes, resulting in a temporary worsening of symptoms [1,8,9,17]. A retrospective review on 3-year off-label use of DIS for Lyme disease treatment on 67 patients demonstrated a significant difference in the incidence of adverse reactions proportional with applied dose by examining the high-dose (>4 mg/kg/day) and low-dose (<4 mg/kg/day) patients [9].

Although oral DIS has been successful in treating alcoholism, its clinical outcomes in other areas like cancer, infectious diseases, and neurological disorders remain limited due to poor solubility, instability, low bioavailability, rapid metabolism, and inefficient tissue targeting [19,20]. Effective repurposing requires addressing both clinical and technological challenges [18]. Nanoscale drug delivery systems, particularly cyclodextrins (CDs), offer significant advantages by overcoming PK and formulation barriers. Compared to other nanocarriers, CDs provide simpler, reproducible molecular encapsulation, enhancing delivery efficiency, reducing toxicity, and enabling multifunctional and non-invasive delivery [18,20]. CD-based formulations improve solubility, stabilize the drug, and support improved release, addressing key pharmacological limitations that have hindered DIS’s broader clinical use [18].

The present drug delivery system development strategy seeks to significantly improve the treatment profile of the repositioning drug in Lyme disease. By leveraging CD derivates and formulating rectal suppositories, this pharmaceutical formulation aims to reduce the necessary DIS dosage compared to oral tablets, to increase the solubility of the drug and the bioavailability by avoiding the hepatic first-pass effect, and to minimize dose-limiting toxicity.

## 2. Materials and Methods

### 2.1. Materials

DIS (Mw = 296.54 g/mol, purity 97%) was obtained from Sigma-Aldrich Chemie GmbH (Schnelldorf, Germany). The CD derivatives, hydroxypropyl-β-cyclodextrin (HPBCD), degrees of substitution (DS)∼4.5, (Mw = 1387), and randomly methylated-β-cyclodextrin (RAMEB) DS∼12 (Mw = 1303 g/mol) were the products of Cyclolab R&D Ltd. (Budapest, Hungary). Ethanol was purchased from Molar Chemicals Ltd. (Halásztelek, Hungary), and methanol, acetonitrile, n-dodecane; the salts used to make the buffer were ordered from Sigma-Aldrich Hungary (Budapest, Hungary). Ultrapure, deionized water was prepared by a Milli-Q Direct 8 Millipore system (Milford, MA, USA). All other chemicals used were of analytical grade from commercial suppliers. Hard fat (HF, synonyms: Adeps solidus 50, Witepsol W35), polyethylene glycol (PEG, synonym Macrogola) 1500 and 4000 were kindly provided by University Pharmacy Department of Pharmacy Administration, Semmelweis University (Budapest, Hungary). Acceptor Sink Buffer and microFlux polyvinylidene difluoride (PVDF) hydrophobic membranes were purchased from Pion Inc. Ltd. (East Sussex, UK). Torpedo-shaped plastic disposable suppository molds (capacity of 3 g) were obtained from Pharmacy-Technology Ltd. (Miskolc, Hungary). Hydrophilic PTFE syringe filters, with 25 mm diameter and 0.45 µm pore size were ordered from Lab-Ex Ltd. (Budapest, Hungary).

### 2.2. Preparation of Inclusion Complexes and the Respective Physical Mixtures

Inclusion complexes of DIS were prepared with HPBCD or RAMEB in 1:2.5 (DIS:CD) mole ratio with the co-dissolution method, adopted from previously described techniques [30]. Briefly, CDs and DIS were separately dissolved in ethanol, then solutions were mixed under continuous stirring for 24 h at 25 °C. The solvent was removed by evaporation, using rotary evaporator (BÜCH Rotavapor R-200, Flawil, Switzerland) on 40 °C, 20–25 min. The physical mixtures were prepared at the same molar ratio in a ceramic mortar.

### 2.3. Preparation of the Suppositories

The suppositories were prepared by melting and molding, using different hydrophilic (PEG blend) and lipophilic (HF) suppository vehicles. The bases were melted by means of an infrared lamp, just about a few degrees above their melting point, taking care not to reach 70 °C. DIS, inclusion complexes, or the physical mixtures of DIS and CDs were added to the melted bases in small amounts with constant stirring, which were previously passed through a sieve with a mesh size of 0.16 mm. The mixture was poured into disposable plastic conical molds and allowed to cool at room temperature (15–25 °C). Suppository molds were previously calibrated for each suppository base and composition, using the double pour method.

### 2.4. Dissolution of Suppositories

Dissolution tests were performed with Agilent Varian VK7020/7025 (Agilent Technologies, Inc., Santa Clara, CA, USA), using 900 mL phosphate-buffer saline (PBS) pH 7.4 medium, at 37.4 °C. The suppositories were placed in baskets specially designed for the dosage form and agitated at 75 rpm. Drug content was determined using a UPLC method with the following parameters: column—Waters Acquity UPLC BEH C18 (1.7 µm, 2.1 × 50 mm); mobile phase—isocratic 70:30 methanol:water; column temperature—40 °C; sample temperature—25 °C; injection volume—1 µL; flow rate—0.6 mL/min; run time—1 min; detection—270 nm. The calibration curve is set up from methanol solutions of DIS in 5–35 μg/mL dose range. For each measurement, three replicates were used (*n* = 3).

### 2.5. Dissolution Profile Comparison—Statistics and Analysis of the Similarity Factor, f_2_

Comparison of the dissolution profile was performed using a model independent method by determining the similarity factor (f_2_). In designing the statistical analysis, the internationally harmonized f_2_ criteria system recommended for dissolution comparison was followed (*n* = 12) [31]. The dissolution profile comparison studies were performed using OriginPro 2022 software (OriginLab Corp., Microcal Software, Inc., Northampton, MA, USA), for which the Drug Dissolution Analysis extension, available for download from the software developer’s website, was used. In order to measure the dissimilarity between the mean dissolution profiles of the samples by accounting for the correlations and variances across multiple time points in the dataset, Mahalanobis distance was determined.

### 2.6. Small Volume Dissolution–Permeation Analysis

The permeability of DIS containing suppository samples were tested using μFLUX (Pion Inc., Billerica, MA, USA) apparatus, which consists of a donor and an acceptor chamber separated by an artificial membrane (PVDF, 0.45 μm, 1.54 cm^2^) impregnated with 25 µL n-dodecane to form a lipophilic barrier between the donor and acceptor chambers. The acceptor chamber, representing blood circulation, was filled with 20 mL Acceptor Sink Buffer (ASB, Pion Inc., Billerica, MA, USA). For the donor chamber, 20 mL of pH 7.4 PBS was prepared. The 30 mg DIS-containing suppositories were remelted in an ultrasonic bath thermostated at 60 °C. The melt was rapidly poured into a silicone mold using a pipette. The solidified samples were weighed one by one, and the average mass and the standard deviation were determined. By comparing the mass of each sample with the original mass of the suppository, the theoretical amount of DIS was calculated, corresponding to 1 mg on average in each split sample. A split sample containing 1 mg of the drug was applied for measurement, i.e., an equivalent to 50 µg/mL of DIS was placed in the donor chamber containing 20 mL media, and both chambers were stirred with a magnetic stirrer (speed 250 rpm) at 37 °C. To evaluate the concentration of DIS, the calibration was conducted using the appropriate tips (5–20 mm pathlength) adjusted to the fiber optic UV dip probe. The calibration was performed using 15 mg/mL and 5 mg/mL methanol stock solutions of the analyte for each UV dip probe separately, adding an equivalent amount of this solution to all 6 vessels, containing PBS (supplemented with 20% HPBCD) or ASB, and registering the UV spectrum after each step. From the known concentration values and UV data, the calibration curve was determined with linear regression (R^2^ ≥ 0.9990 in each case). 5 mm tips were used on the donor side for sample suppositories containing DIS, CDs, and PEG, and calibrated in 15–75 µg/mL dose range. 20 mm tips were used for the reference suppositories, containing only DIS with PEG, and for the acceptor side, and were calibrated in 5–25 µg/mL dose range. The evaluation was performed using the second derivative method within the range of 304–320 nm in the donor side and between 302 and 320 nm in the acceptor side. The UV spectra were registered as follows: 12 spectra per 5 s in the first minute, then 1 spectrum per 30 sec, with different experiment times. The flux and permeability values were calculated using Equations (1) and (2) between 20 and 40 min. The flux (J) across the membrane was calculated using the following equation:

(1) $$J\left(t\right)=\frac{\Delta n}{A\times \Delta t}$$

where the flux of a drug through the membrane is defined as the amount (n) of drug crossing a unit area (A) perpendicular to its flow per unit time (t); from this, the effective permeability (P_eff_) of the drug is deducted as follows:

(2) $$P_{eff}=\frac{J}{C_{D}}$$

where C_D_ is the donor concentration [32].

### 2.7. Quality Control of Selected Suppository Composition

#### 2.7.1. Weight Uniformity

To evaluate the uniformity of weight, 20 suppositories were randomly selected and weighed. Uniformity of weight was determined based on the acceptance criteria: none of the suppositories should be weigh ±5% of the average weight of the suppositories.

#### 2.7.2. Content Uniformity

The UV/VIS spectroscopy method was adopted for the assay of rectal preparations [33]. The drug content of selected composition suppositories containing 30 mg DIS was determined with a Jasco V-750 UV/VIS spectrophotometer with 10 mm quartz cuvettes at 25 °C. The active substance sampling was conducted by dissolving each suppository (*n* = 10) in 100 mL distilled water at 37 °C for 120 min on a magnetic stirrer with continuous stirring, filtering through syringe filters (0.45 µm pore size), and diluting 200 µL filtered stock solution to 10 mL with methanol, using the solvent as blank solution. The external standard method was applied to determine DIS concentrations. The standard stock solution was made with 100 µg/mL DIS content in methanol, then diluted with the solvent to set up the calibration curve (y = 0.046x − 0.0327, R^2^ = 0.999) with standards of DIS in the 2–8 µg/mL dose range. The absorbances were measured at 275 nm for each sample. The content uniformity test complies with the acceptance criteria if no more than one individual content is outside the limits of 85% to 115% of the average content and none are outside the limits of 75% to 125% of the average content.

#### 2.7.3. Disintegration

The hydrophilic suppositories were subjected to 60 min disintegration test, carried out on ERWEKA ST 35 (Erweka GmbH, Langen, Germany) with three turnable test stations (*n* = 3). The test stations were placed in a thermostatically heated water bath. Distilled water (37 ± 2 °C) was used as an immersion fluid (4 L), and the baskets with the samples were immersed in the fluid, stirred with low and a constant frequency rate and automatically turned by 180° at 10 min intervals.

#### 2.7.4. Hardness

Three randomly selected samples of the suppository formulation were analyzed for hardness using a Dr. Schleuniger Pharmatron Tablet Hardness Tester, Model 8M (Pharmatron AG, Thun, Switzerland). Each suppository was kept between two plungers of the hardness tester and pressure was applied until the suppository deformed or snapped.

#### 2.7.5. One-Month Drug Content Stability Monitoring with UV/VIS Method

The UV/VIS spectroscopy and sampling method employed for content uniformity assessment were used to evaluate changes in drug content over a one-month stability test. Concentrations were analyzed weekly from week 0 to week 4 (*n* = 3 for each time period). The suppositories were packed in plastic disposable molds covered by white polyethylene self-adhesive strip, using cardboard boxes as secondary packages, and were stored in a programmable temperature and humidity test chamber at a controlled condition of 25 °C and 60% relative humidity (RH).

#### 2.7.6. Differential Scanning Calorimetry (DSC)

Prior to analysis, the suppositories were grated, and the raw materials were mashed in a mortar and pestle to create a uniform blend. The samples were measured into a 40 µL pierced aluminum pan with weight of 10 ± 0.5 mg, then a traditional linear heating DSC method (DSC3+ device, Mettler Toledo AG, Zürich, Switzerland) was applied from 25 °C to 170 °C with a heating rate of 10 °C/min. The DSC chamber was purged with dry nitrogen using a flow rate of 50 mL/min.

#### 2.7.7. X-ray Diffraction Study (XRD)

Suppository formulations, inclusion complexes and physical mixtures of DIS, CDs, and the vehicles with the same ratio as the corresponding suppository, were tested. The sample preparation method used for DSC was applied here as well. XRD patterns were obtained using a PANalytical Empyrean diffractometer and data analysis was conducted with HighScore program (Malvern Panalytical Ltd., Malvern, UK). X-ray radiation was produced by a copper X-ray tube with a wavelength of 1.541874 Å (Cu Kα) and was focused by a focusing elliptical mirror. Accelerating voltage and anode heating current values were set to 45 kV and 40 mA, respectively. Silicon powder was used as line position and line shape standard and alumina plate was applied as relative intensity standard (both standards are certified, originated from National Institute of Standards and Technology, NIST). The instrument was used in transmission mode and the samples were placed into the sample holder between two Mylar foils without grinding. Samples were rotated (1 rps) during the measurement. A PIXcel 3D 1 × 1 area detector in scanning line detector (1D) mode was used at the diffracted side. Measurements were carried out with a step size of 0.01°2θ, a measurement range of 2.00–50.00°2θ, and a time per step value of about 335 s. The whole process was carried out at room temperature over about a 85 min/sample period.

#### 2.7.8. Attenuated Total Reflectance Fourier-Transform Infrared Spectroscopy (ATR-FTIR)

The infrared spectra were used for comparison of the primary materials, previously recorded reference spectra, and suppository spectra. The ATR-FTIR spectra were determined by a Jasco FT/IR-4600A instrument with ATR Pro ONE accessory (JASCO Ltd., Tokyo, Japan) over the range of 400–4000 cm^−1^ at a resolution of 4 cm^−1^. A deuterated triglycine sulfate detector was used, and 64 scans were accumulated for each composition. Background spectra were recorded and subtracted from the samples.

## 3. Results

### 3.1. Preparation of Suppositories

**Figure 1:**
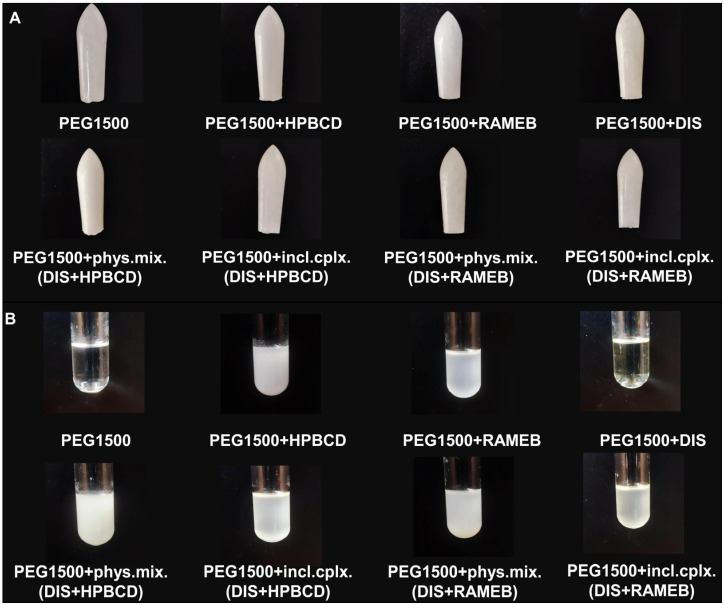
Picture of (**A**) suppositories and (**B**) their melts. Abbreviations: DIS = disulfiram, HPBCD = hydroxypropyl-β-cyclodextrin, incl.cplx. = inclusion complex, PEG = polyethylene glycol, phys.mix. = physical mixture, RAMEB = randomly methylated-β-cyclodextrin.

The preparation of inclusion complexes and their characterization was previously published [30]; to ensure the formation of inclusion complexes for each batch of production, screening XRD and DSC methods were applied (Figure S1). Each suppository contained 1% DIS and 11% HPBCD or RAMEB (*w*/*w*). The active substance content per rectal suppository was determined from literature sources, based on published treatment protocol [8], using a starting dose of 1/16 of a 500 mg tablet administered every three days at the start of therapy to reduce the risk of Herxheimer reactions, thus each suppository was designed with 30 mg DIS content. The amount of CDs was calculated according to a 1:2.5 mole ratio DIS:CD, as previously determined [30], and to EMA/CHMP/333892/2013 perspectives. In addition to the sample rectal suppositories, blank (without DIS) and reference (without CDs) rectal suppositories were tested as controls and to set up the analytical method. When blank suppositories containing only CD and suppository vehicle were prepared, it was observed that a suspension was formed by the CDs with the melt, whereas in the case of the reference sample containing only DIS in the vehicle, it was seen that the active ingredient dissolved in the melt. The suppositories of inclusion complexes and physical mixtures of DIS and CDs form a suspension with the melt too (Figure 1). Due to the presence of two phases, we measured the particle size and size distribution of raw materials (Figure S2) [33], and to ensure size limiting accuracy, the powders were passed through the sieve.

### 3.2. Dissolution Test-Driven Composition Optimization

**Figure 2:**
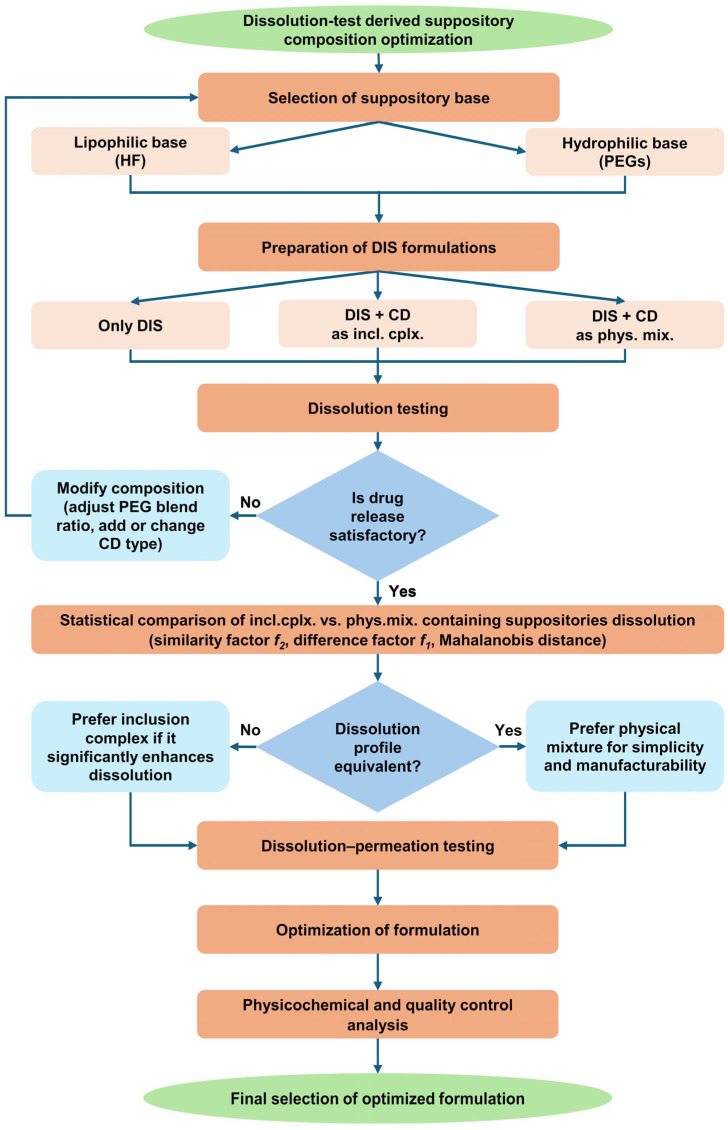
Flow chart of dissolution test-driven DIS containing suppository composition optimization. Abbreviations: CD = cyclodextrin, DIS = disulfiram, HF = hard fat, incl.cplx. = inclusion complex, phys.mix. = physical mixture, PEG = polyethylene glycol.

The release of an active substance from a rectal suppository is an important factor in the transmucosal absorption of drugs because the rectal fluid has a small volume compared to gastrointestinal fluid and the rectum has a relatively small surface area compared to the small intestine [34,35]. Therefore, the dissolution of the rectal suppository in a biorelevant medium was chosen as a screening method for formulation optimization (Figure 2).

#### 3.2.1. Suppository Base Selection

**Figure 3:**
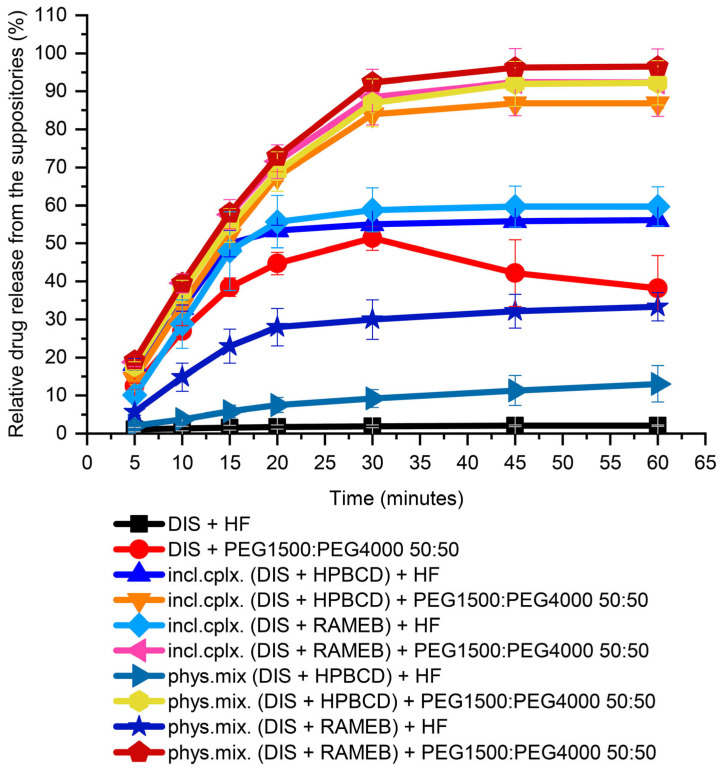
Dissolution test of DIS-containing suppository samples for base selection. Abbreviations: DIS = disulfiram, HF = hard fat, HPBCD = hydroxypropyl-β-cyclodextrin, incl.cplx. = inclusion complex, PEG = polyethylene glycol, phys.mix. = physical mixture, RAMEB = randomly methylated-β-cyclodextrin.

In preformulation, both water-insoluble lipophilic bases (HF) and water-soluble hydrophilic blends (PEGs) were tested for choosing a suppository carrier. For the first screening from PEGs, a 50:50 mass ratio of PEG1500 and PEG4000 blend was used. From HF, about 2% of the 30 mg DIS was released in 60 min. From the PEG blend, 50% relative release was achieved in 30 min, but the active substance had precipitated over time and equilibrated about 40% at the end of measurement, showed by the breakpoint of the dissolution profile. In the presence of CDs from the lipophilic carrier, only a maximum of 50% release of the active substance was achieved, while the hydrophilic carrier could provide release above 80% (Figure 3). From the PEG blend, the dissolution of the drug from the inclusion complex or from the physical mixture with CDs has not deviated significantly. In contrast the release from HF of inclusion complexes were approximately twice as high as from the physical mixture. This could be attributed to the hydrophobicity of raw DIS, showing affinity to the lipophilic phase formed by the HF. Through CD encapsulation, the solubility of the Biopharmaceutical Classification System (BCS) II class drug is enhanced, and the inclusion complexes provide a faster dissolution than the physical mixtures [30]. For a host–guest interaction to occur, there must be a favorable net energetic driving force that pulls the drug molecule into the CD cavity, and the final equilibrium takes longer time to attain [36]. Moreover, in the lipophilic base, a difference between the CDs physical mixtures could be seen; RAMEB provided a more favorable drug release than HPBCD (33 ± 3.7% and 13 ± 4.8%, respectively), which converges with the equilibrium constant of the complexes with DIS [30]. For the further drug development, PEGs were selected as suppository vehicles; however, the melting point of solid PEGs, in general, is higher than of lipophilic bases, which melt at body temperature [37].

#### 3.2.2. Investigating the Effect of Different PEG Blends on Formulation

**Figure 4:**
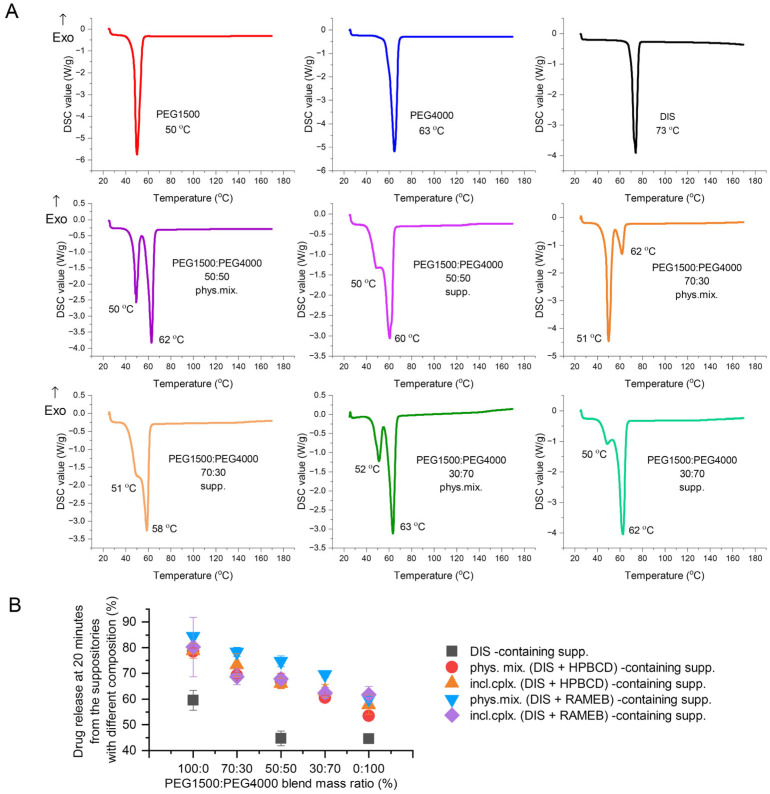
Investigating the effect of different PEG blends on formulation: (**A**) Differential scanning calorimetry (DSC) analysis of PEG1500:PEG4000 blends prepared by physical mixing of the components in respective mass ratio or the co-melting, molding, and grating of suppositories. (**B**) Drug released at 20 min from the suppositories of different compositions, made with PEG1500 and PEG4000 blends in various mass ratios. Abbreviations: DIS = disulfiram, exo=exothermic thermal event direction, HPBCD = hydroxypropyl-β-cyclodextrin, incl.cplx. = inclusion complex, PEG = polyethylene glycol, phys.mix. = physical mixture, RAMEB = randomly methylated-β-cyclodextrin, supp. = suppository.

The active pharmaceutical ingredient’s relatively low melting point (70 °C), lipophilic nature (logP = 3.88), and poor water solubility (4 mg/L) are critical parameters to consider [30,38,39]. Therefore, the primary goals of preformulation are to ensure DIS’s thermostability during production and to increase its solubility, using different blends of PEG1500 and 4000. The thermo-analytical technique, DSC, was applied for melting point determination; the drug release tests were carried out for the optimal PEG blend selection, ensuring maximized dissolution and minimized drug instability. The comfort temperature gap between DISs and different compositions of PEG1500 and PEG4000 carriers was found with only PEG1500-containing samples, presenting the largest difference between the melting point of the active ingredient (73 °C) and the suppository base (50 °C) (melting point difference > 20 °C) (Figure 4A). The dissolution-based comparison of DIS containing suppositories with PEGs and blends was evaluated at 20 min, when none of the release profiles reached the plateau phase. Decreasing the mass ratio of PEG1500 in carrier blends, the dissolution values also showed a declining trend (Figure 4B). The production of rectal suppositories uses melting and molding technology, therefore the selection of 100% PEG1500 as a carrier is to be considered to prevent thermal degradation, and to achieve a preferred drug release.

#### 3.2.3. Dissolution-Driven Justification of the Preliminary Inclusion Complex Preparation Step in the Suppository Formulation Process

The physicochemical properties of the free drug molecules and the free CDs are different from their counterparts in the complexed form [36], thus the study of physical mixtures was considered justified alongside the DIS and CD inclusion complexes during the formulation of the suppositories. The advantage of inclusion complex formation is indisputable, as CDs improve the bioavailability of the drug through increases in aqueous solubility and dissolution rates [30,36]. This could be clearly seen in the case of the samples with HF vehicle; suppositories containing inclusion complexes provide a double release rate of the drug in contrast to physical mixture-containing ones. However, for PEG-based suppositories, this evident difference disappears from the dissolution profiles (Figure 3), thus statistical analysis was applied to evaluate the similarity of the dissolution profiles. The model-independent similarity factor (f_2_) approach is a relatively simple and widely accepted method for comparing dissolution profiles, and is required by regulatory authorities [31]. The inclusion complex-containing samples were named as the reference, the physical mixture containing suppositories formed the test group. The comparison analysis applied the recommended f_2_ harmonized criteria system by Diaz et al. [31]. Testing was conducted under identical conditions using 12 dosage units for both test and reference suppositories. Five sampling time points were selected to characterize the dissolution profiles, at 5, 10, 15, 20, and 30 min, distributed between the ascending and inflection portions of the profile, with only one point considered at 30 min closer to the plateau of the curve and after 85% dissolution of the test and reference. The percentage coefficient of variation at the earlier time points (<15 min) were not more than 20%. The criteria for the other time points to not be more than 10% were not achieved, but not being more than 15% at all time points were accomplished. The f_2_ factor is a logarithmic reciprocal square root transformation of the sum of squared error and is a measurement of the similarity in the percent dissolution between the two profiles [31]. The difference factor (f_1_) calculates the percent difference between the two dissolution profiles at each time point and is a measurement of the relative error between the two profiles [31]. f_1_ values up to 15 (0–15) and f_2_ values greater than 50 (50–100) ensure the “sameness” or “equivalence” of the two profiles [31]. The dissolution profiles comparison involves calculating the Mahalanobis distance between the mean dissolution profiles in n-dimensional space, where n is the number of dissolution time points in the dataset [31]. The test and reference samples can be considered to have similar profiles if the upper limit of the confidence interval calculated between the reference and test sample is less than or equal to the similarity limits derived from testing multiple reference batches. The dissolution profiles of the inclusion complexes and physical mixtures were similar (Table 1 and Figure S3), presuming a synergism of PEG and CDs on DIS dissolution. 

#### 3.2.4. CD Type Selection

The similarity study of the release profile of the rectal suppositories prepared with the two types of CDs (HPBCD or RAMEB) were conducted by contrasting the inclusion complexes between them [incl. cplx. (DIS + HPBCD) + P1500:P4000 50:50] versus [incl. cplx. (DIS + RAMEB) + P1500:P4000 50:50] and physical mixtures among themselves [phys.mix. (DIS + HPBCD) + P1500:P4000 50:50] versus [phys.mix. (DIS + RAMEB) + P1500:P4000 50:50], using the same harmonized criteria system [31] (Figure S3). f_2_ values above 50 of the physical mixtures—68.10 ± 5.69 and for inclusion complexes 61.11 ± 2.76—suggest that the quality of CD does not significantly influence the release rate of DIS.

### 3.3. Results of Small Volume Dissolution–Permeation Analysis

**Figure 5:**
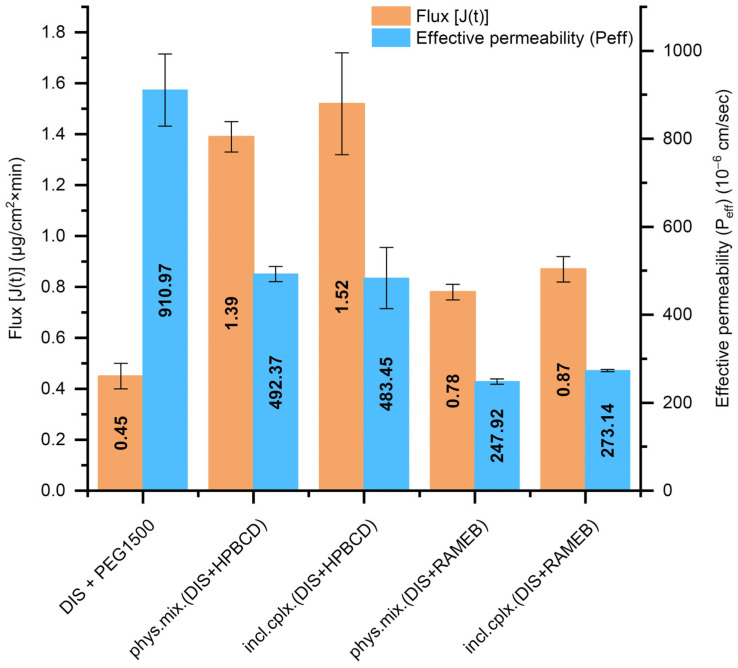
T The flux [J(t)] across the artificial membrane and the effective permeability (P_eff_) values of DIS from the measured suppository samples calculated between 20 and 40 min. Abbreviations: DIS = disulfiram, HPBCD = hydroxypropyl-β-cyclodextrin, incl.cplx. = inclusion complex, PEG = polyethylene glycol, phys.mix. = physical mixture, RAMEB = randomly methylated-β-cyclodextrin.

The DIS-containing PEG suppositories’ dissolution profile equivalency with inclusion complexes or physical mixtures and the use of different CD types were further analyzed. Thus, the Pion MicroFLUX device was applied, combining dissolution experiments with transmembrane permeation. The in situ fiber optic UV monitoring system allows us to monitor the drug’s concentration in real-time, simultaneously from the small-volume acceptor and the donor chamber separated with a biomimetic lipid permeation barrier [32]. The exclusively DIS-containing PEG suppositories presented the highest effective permeability (P_eff_, Figure 5); however, the flux-profile [J(t), Figure 5] through the artificial membrane was under 0.5, whereas in the presence of HPBCD, nearly threefold more molecules went through the membrane, primarily due to the markedly higher amount of dissolved drug facilitated by the solubilizing effect of the CDs. Suppositories with HPBCD exhibited approximately twice the permeability of the formulations with RAMEB. The difference in flux and permeability profile between the inclusion complex and the physical mixture was negligible at the level of both CDs (Figure S4). Based on dissolution and permeation results [phys. mix. (DIS + HPBCD) +PEG1500], suppositories were selected for further physico-chemical analysis.

### 3.4. Results of Quality Control on Selected Suppository Composition

All the suppositories were white to slightly yellowish in color and had a smooth and even surface without any visible cracks. The average weight was 3.14 ± 0.03 g and the weight variance measured for 20 suppositories was determined to be under ±1.5%, in accordance with the criteria European Pharmacopoeia (Ph. Eur.) requirements for the Uniformity of Mass of Single-Dose Preparations (Ph. Eur. 2.9.5); no more than 2 of the individual masses deviated from the average mass by more than a 5% percentage deviation, and none deviated by more than twice the percentage. The uniformity of drug content was determined on 10 individual suppositories, and the individual contents were within limits (85–115%) set with reference to the average content of the sample, presenting a mean of individual contents of 99.5%, standard deviation 2.95, relative standard deviation 3, and acceptance value 7.1, calculated with 2.4 acceptability constant. In water, the PEG suppositories disintegrated and dissolved within 29.59 ± 0.63 min. The suppositories showed good mechanical strength, 211.6 ± 4 N, and ideal hardness needed for handling, storing, and transporting. After one month at 25 °C, no color change was observed in the suppositories. The weekly UV/VIS monitoring of drug content showed no significant variation in the DIS load of the suppositories (Table 2), thereby confirming the stability of the formulation over one month. Additionally, the DIS content’s weekly variations and content uniformity deviations were similar.

#### 3.4.1. DSC

**Figure 6:**
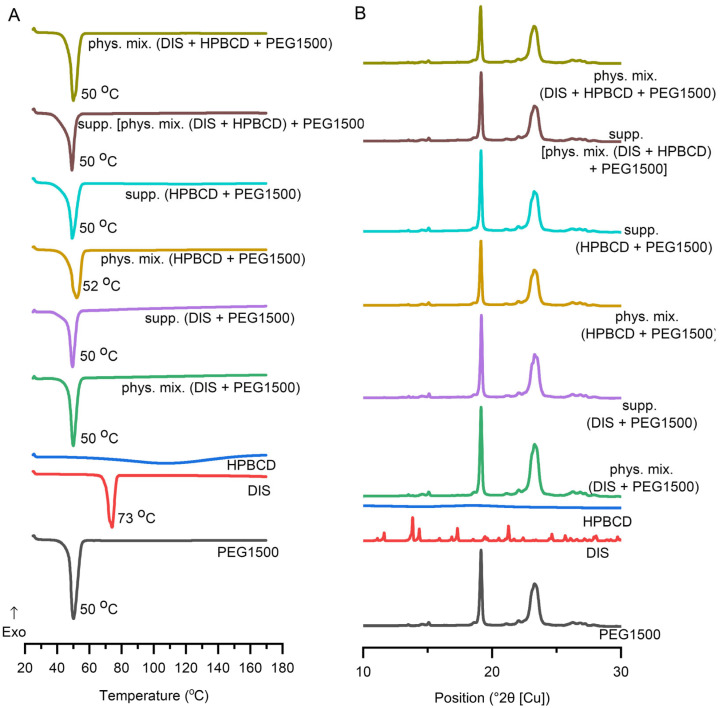
Characterization of suppositories with (**A**) differential scanning calorimetric (DSC) analysis, and (**B**) X-ray diffraction. Abbreviations: DIS = disulfiram, exo=exothermic thermal event direction, HPBCD = hydroxypropyl-β-cyclodextrin, PEG = polyethylene glycol, phys.mix. = physical mixture, supp. = suppository.

DSC was applied to determine the interaction between the DIS and the excipients and to ascertain the impact of the production process. In addition to the raw materials, selected suppository and respective physical mixture, the characterization expanded on the binary system of the components. DIS and HPBCD physical mixture and their inclusion complexes are described in the literature [30,39,40,41,42,43], thus monitoring DSC and XRD analysis was applied (Figure S1). DIS showed a characteristic sharp melting endothermic peak at 73 °C, and PEG1500 at 50 °C. In contrast, HPBCD lacks sharp melting endotherms of crystalline drugs, confirming the amorphous state of CDs and the broad endothermic peaks in the 50–150 °C range representing the water loss. The suppository and physical mixture of the base and the drug and/or CD have shown similar endothermic peaks at about 50 °C, corresponding to the melting point of PEG1500. The characteristic peak of DIS is absent from the thermograms of suppositories and respective physical mixtures, suggesting its solubilization in the melt of PEG1500 even in situ during the analysis (Figure 6A), explicable with the low drug load. The disappearance of a drug’s crystalline structure during the applied melting and molding suppository production technology involves the complex molecular interactions and dissolution behavior of the drug in the excipient environment, seen also in the dissolution profile difference of suppository samples containing only DIS and PEG1500 or DIS, HPBCD, and PEG1500.

#### 3.4.2. XRD

DIS and PEG1500 exhibit sharp, high-intensity diffraction peaks in its XRD pattern, indicating a highly crystalline structure. HPBCD exhibits a broad, featureless background in XRD, reflecting its amorphous structure. All the suppository samples and respective physical mixtures presented a pattern like PEG1500. The formulated DIS showed reduced crystalline peaks (Figure 6B), however the characteristic peak of DIS at position 13.8 (°2θ [Cu]) is detectable in all the DIS-containing samples (Figure S5). The fading pattern of the drug in physical mixtures and suppository samples might be explained by the composition of the blend, containing only 1% (*w*/*w*) of DIS.

#### 3.4.3. FTIR

**Figure 7:**
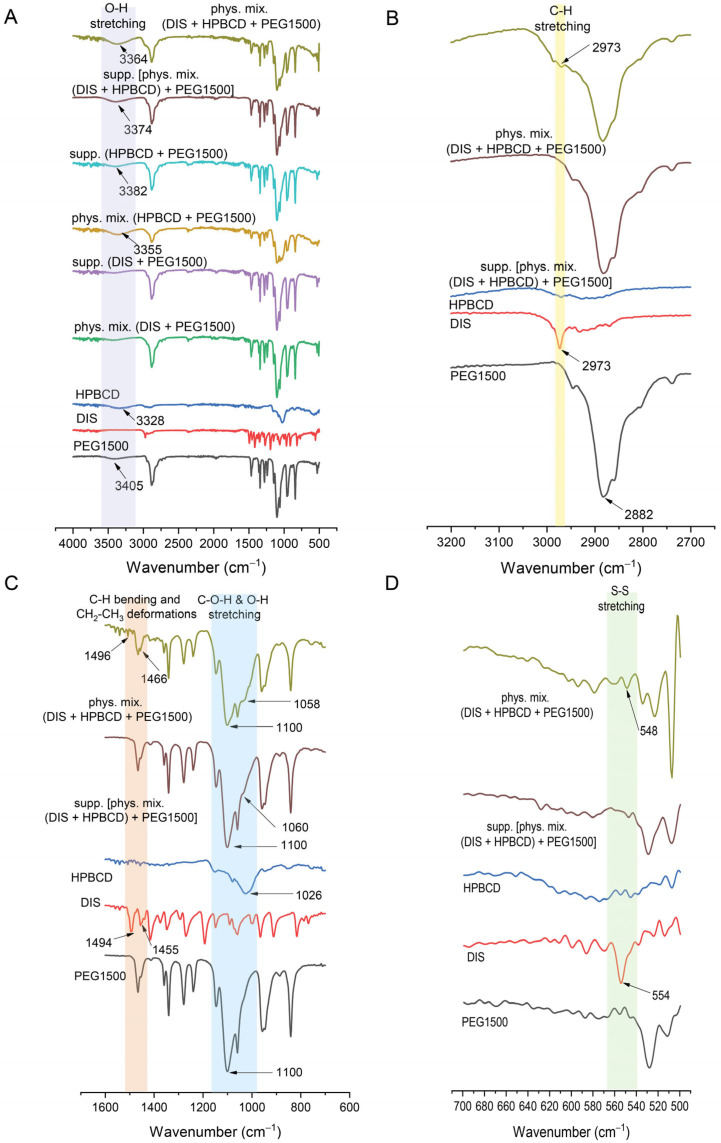
Fourier transform infrared spectroscopy (FTIR) of suppositories in range of (**A**) 400–4000 cm^−1^, (**B**) 2700–3200 cm^−1^, (**C**) 600–1600 cm^−1^, (**D**) 500–700 cm^−1^. Abbreviations: DIS = disulfiram, HPBCD = hydroxypropyl-β-cyclodextrin, PEG = polyethylene glycol, phys.mix. = physical mixture, supp. = suppository.

FTIR offered a valuable tool in the characterization of suppository samples in contrast to physical mixtures, providing insights into the molecular structure and showing the shifting in frequency of some bonds with respect to control spectra of PEG1500, DIS, and HPBCD (Figure 7A). The spectrum of DIS showed the characteristic vibrational peak at 2973 cm^−1^ assigned to C-H stretching, which was detectable also in physical mixtures (Figure 7B). The peak of DIS at 1494 cm^−1^ attributed to C-H symmetrical deformation vibrations, the peak at 1455 cm^−1^ assigned to CH_2_-CH_3_ deformations (Figure 7C), and the peak at 554 cm^−1^ assigned to S-S stretching (Figure 7D) were shifted to 1466, 1496, and 548 cm^−1^ in physical mixtures. The characteristic peaks of DIS may be slightly detected in the FTIR spectra of the rectal dosage form; the PEG1500 base spectra overlapped the drug’s characteristic peaks. The identified spectrum and the typical peaks of the drug are in accordance with the literature data [42,44]. In the case of HPBCD and PEG1500, the broad absorption band around 3400 cm^−1^ is indicative of O-H bonding (Figure 7A) [45]. The O-H stretching is detectable at about 3405 cm^−1^ in PEG1500 and 3328 cm^−1^ in HPBCD, which are shifted to 3355 and 3364 cm^−1^ in physical mixture controls and 3382 and 3374 cm^−1^ in suppository samples. Peaks at approximately 2900 cm^−1^ are associated with aliphatic C-H bonds in PEG1500 (Figure 7B). The characteristic absorption peak of PEG1500 at 1110 cm^−1^ is attributed to the stretching vibration of C-O-H and O-H bond [46], whereas in the case of HPBCD, this frequency is shifted from 1026 cm^−1^ to 1060 cm^−1^ in suppository and to 1058 cm^−1^ in physical mixture (Figure 7C).

## 4. Discussion

### 4.1. Low-Dose DIS in the Treatment of Lyme Disease

DIS is a promising non-antibiotic alternative adjuvant therapy in treating persistent symptoms, enduring 6 months or longer, after initial Lyme disease treatment; as a repositioning drug it is considered safe and well-tolerated, enhancing the quality of life in patients [1,9]. All unicellular organisms require Zn^2+^, Mn^2+^, and Cu^2+^ as central cations for activity of enzymes in mitochondria (superoxide dismutase: Mn^2+^, ALDH2: Zn^2+^). Withdrawing these atoms by metal chelation with DIS blocks the mechanism for energy supply (ATP) and replication which ultimately leads to the death of these microorganisms and is obviously the reason that low dosages work selectively on microorganisms. On this mechanism of action is based the cutaneous emulsion Tenutex^®^, containing 2% DIS in combination with 22.5% benzyl benzoate, which is a prescription free drug in Sweden that is active against scabies, head lice and pubic lice [19,47]. The development of a rectal suppository containing 30 mg DIS was governed by the existent literature data on benefits and risks of DIS use in Lyme treatment, endeavoring to achieve a low dose to minimize the incidence of adverse actions and to avoid hepatic first-pass effect and to maximize the drug’s bioavailability in the new indication area. According to Gao et al., dosages as low as 0.06–2 mg/kg/day for indeterminate durations conferred benefit with minimal adverse effects [9,29]. The drug content of the rectal formulation is in accordance with low dose regimen recommendations and with DIS therapy initiation and management perspectives [8,9]. In human off-label use, DIS was associated with side effects, such as Herxheimer reactions at the initiation of therapy and, of greater concern, neurotoxic reactions, typically occurring at higher doses of DIS, but if the dose was lowered or if the drug was stopped within days of the onset of neurotoxic symptoms, they usually remitted quickly [8,11,17]. Off-label use of medicinal agency-approved drugs is within the discretion of practitioners, considering that DIS is useful in treating persistent symptoms after initial Lyme disease treatment, and is under condition of personalized and upscaling dose regimen, regular laboratory monitoring, and close clinical follow-up, preventing neuro-, and liver toxicities [1,8,9,10,11,17,24,27,28,48]. Although the in vitro and preclinical findings of DIS to possess potency against *B. burgdorferi* and the clinical reports are encouraging, there is still a need for more pharmacological studies and randomized controlled clinical trials to clarify its translational potency in Lyme disease [49].

### 4.2. Role of Suppository Matrix

The release of the active ingredient from the lipophilic suppository matrix was far below that of the hydrophilic base. The selected vehicle (PEG1500) is a conventional suppository base, presenting the advantage of relatively large difference (20 °C) between its melting point and of the active ingredient, providing the drug’s thermostability during the suppository formulation. The rapid, unwanted metabolism of DIS in the liver leads to its poor delivery efficiency to other tissues, which is a key issue factor in its clinical translation in other indication areas than chronic alcoholism [20]. To overcome the multiple translational barriers, in newly defined indications of DIS [7,20,50], novel drug delivery systems and various carrier materials have been extensively explored which increase the aqueous solubility, protect the rapid degradation in the blood circulation, increase the PKs property of DIS, target delivery of drug moiety in a tissue or cell and, enhance the therapeutic efficacy of encapsulated molecules, such as inorganic nanomaterials, polymeric nanoparticles, liposomes, and CDs [20,50]. The use of CD inclusion complexes to enhance the solubility of DIS is reported in the literature for anticancer [18,30,39,41], SARS-CoV-2 [42], ophthalmological [40,43], addiction indications (WO2009083793A1), and in Lyme disease [13]. CDs favor solubility protect the drug from chemical and enzymatic degradation, limit the toxic effects in some instances, and act as penetration enhancers [30,36,51]; combining the pharmaceutically relevant CDs and the already approved chemical entity is advantageous from drug repositioning strategy perspectives. The presence of CDs enhanced the drug’s release from both lipophilic and hydrophilic suppository carriers in the biorelevant media. The type of CD excipient and the quantity used was adjusted to the recommended CD dosage threshold values, toxicology, and to the complexation efficiency characteristics; thus, HPBCD was selected and added in the rectal suppository composition under 12% (*w*/*w*) [52]. Although RAMEB presents a more favorable complexation efficiency with DIS than HPBCD [30], HPBCD offered a more favorable flux and permeation profile than RAMEB. The stability constant describes the strength of an interaction between a drug and a CD [51]. If the binding constant presents a low value, the complex formation is decreased, while the proportion of the free drug in this equilibrium system is increased and, according to its logP, traverses the lipid barrier [30]. The permeability order of inclusion complexes is inversely related to their equilibrium constant values, meaning that lower stability constant values correspond to higher permeability for a lipophilic drug such as DIS [30]. Thus, the HPBCD-containing suppositories present more favorable permeability behavior than RAMEB-containing ones. The present results confirm the suitable impact of CDs on the dissolution–permeation profile of a DIS, with typical low water-solubility and high lipophilicity, and their solubility and permeation enhancer role in the composition of suppositories is indisputable. The advantage of guest–host inclusion complexes over physical mixtures is explained by time-dependent complex formation mechanisms; the initial equilibrium to form a complex is rapid, and the final equilibrium takes longer time to attain, resulting in changes in the physicochemical properties of the components: the free drug molecule and the free CD molecule are different from their counterparts in the complexed form [36]. However, the difference in dissolution and permeation profile between the inclusion complex and the physical mixture was negligible with both CDs, which could be attributed to the PEG suppository base. In the preliminary dissolution study of suppositories with HF base, the profile of physical mixtures differs from the inclusion complexes, explicable with the mentioned time-dependent equilibrium constant evolution, and disappears in the case of dissolution and permeation profile of samples with PEG. One possible explanation for this phenomenon could be that melted PEG dissolves DIS and forms a dissolution, enhancing the synergistic matrix with CDs. In solid dispersions, dissolution occurs in two phases: a carrier-enabled dissolution, when excipients rapidly release drug molecules at low drug loads, and a drug-defined dissolution, when at higher drug loads, the intrinsic solubility of the crystalline drug dominates [53]. This was seen in the case of only DIS-containing PEG1500 base suppositories where the dissolution profile presented a breakdown due to precipitation. In contrast, in the presence of CDs offering transition through an intermediate amorphous phase during solubilization, it prevented the drug particle agglomeration. The precipitation is likely due to the limited solubilizing capacity of PEG when it becomes diluted by the aqueous dissolution medium. As PEG dissolves and mixes with water, its ability to keep the drug dissolved decreases, leading to drug crystallization or aggregation. This results in a lower overall amount of the drug remaining in the solution, which can negatively impact drug availability and absorption. This observation highlights the limitation of using PEG alone for sustained drug release and supports the use of CDs or other solubilizing agents to maintain drug solubility and improve release profiles. The molecular interactions during dissolution among DIS, HPBCD, and PEG1500 are crucial factors in dissolution enhancement. Summarizing the dissolution and permeation outcomes [phys. mix. (DIS + HPBCD) + PEG1500], suppositories were selected for further physico-chemical analysis. The DSC confirmed the dissolution of the active substance in the melt of the suppository vehicle. The XRD confirmed the solid dispersion system of DIS and PEG1500. Formulations with a crystalline carrier and drug are one approach, which show a fast dissolution and thermodynamic stability, in contrast with also solubility enhancing but thermodynamically unstable amorphous solid dispersions [53]. FTIR spectral shifts in C-O-H and O-H bands suggest hydrogen bonding interactions between PEG1500 and HPBCD. This molecular association may facilitate the formation of the DIS-HPBCD inclusion complex. The similar dissolution–permeation profiles observed for both the inclusion complex and the physical mixture in suppositories can be attributed to this phenomenon, where HPBCD enhances DIS solubility through inclusion complexation, while PEG1500 likely supports the process via intermolecular interactions. Wulff and Aldén investigated a study on inclusion complex formation between CDs and drugs in the melt of a carrier like PEG, demonstrating the dependence of complex formation on the competitive interaction between the drug and the polymer, as both act as potential guest molecules [54]. However, β-CD does not form complexes with PEG, which might promote the inclusion reaction for the drug in this type of CD [55]. The ability of water-soluble polymers to enhance the solubilizing effect of CDs in a given dosage forms results from a synergistic effect of the polymer and CD, believed to be due to the formation of co-complexes between drug, CD, and polymer [56,57,58,59].

### 4.3. Applicability of DIS-Containing Rectal Suppositories in Lyme Disease

Based on the drug’s dissolution profile similarity from inclusion complexes and physical mixtures, the preparation process of rectal suppositories can be simplified using physical mixtures, which makes the methodology suitable even for broad compounding pharmacy manufacturability. The presented one-month stability is conformed with pharmacy practice guidelines (e.g., United State Pharmacopoeia <795>), which, in the absence of published stability data specific to the compounded formulation, generally recommend a default beyond-use date of 30 days when stored at controlled room temperature. The simple preparation and easy and quick accessibility to patients provides a pharmaceutical formulation that correlates well with the life cycle of the repositionable drug. Suppositories of DIS are not available on the market, and the compliance of the dosage form is lower than that of tablets. However, the advantage of DIS suppository compared to peroral administration is that the rectal administration is less susceptible to the first-pass effect. Due to the favorable bioavailability of rectal use, the required dose can be reduced, and the risk of adverse effects decreases. Considering the duration of therapy (about 6 months), patient adherence is conceivable, which may be confirmed by further in-use studies. The pharmacology of DIS for Lyme disease treatment remains incompletely understood and requires further elucidation. While emerging clinical reports and pilot studies suggest that DIS may reduce symptoms and potentially target persistent *B. burgdorferi* infection, its precise mechanisms of action, optimal dosing, and safety profile in PTLDS patients are not yet well defined [7,8,9,10,13,19]. DIS’ PKs have mainly been studied following oral doses of 250–2000 mg [19], whereas the proposed 30 mg rectal dose represents a significant deviation from established dosing with limited supporting PK data. DIS is highly lipophilic, widely distributes in adipose tissue, crosses the blood–brain barrier, and undergoes extensive metabolism—primarily reduction to diethyldithiocarbamate, followed by degradation, glucuronidation, and methylation [19]. Rectal administration may partially bypass first-pass metabolism, but does not eliminate these pathways. Methylated metabolites contribute to the irreversible ALDH inhibition, key to DIS’s anti-alcoholism effect; however, S-methylation masks thiol groups critical for metal chelation and antimicrobial activity [18]. Significant inter-individual variability in metabolism and non-linear elimination complicate dose–response predictions [18,19]. The toxicity and interaction profile associated with oral disulfiram (DIS) also applies to rectal administration, as systemic absorption and subsequent metabolism occur irrespective of the route of administration. Consequently, risks such as hepatotoxicity, neurotoxicity, and clinically significant interactions—including the disulfiram–ethanol reaction and interactions with drugs metabolized by cytochrome P450 enzymes—remain pertinent with rectal use [19]. Given that DIS is a potential treatment option for Lyme disease with high social media impact [27], it is important to highlight the limitations of the present research: the drug delivery system development lacks in vivo results on the use of DIS suppositories. The proposed rectal formulation aims to modulate systemic exposure by reducing first-pass metabolism, potentially improving bioavailability or favoring therapeutically relevant metabolites. Although translational data are currently limited, using a traditional pharmaceutical drug formulation technology for development and characterization of DIS rectal suppositories, which are generally available in compounding pharmacies and industry, may overcome a few “deadlocks” [60] of DIS and significantly contribute to the development of pharmaceutical strategies for repurposing DIS in novel therapeutic areas.

## 5. Conclusions

By combining the established chemical entity, DIS, at a low dose (30 mg per suppository) with a safe and effective dose of the pharmaceutical excipient HPBCD, and utilizing a traditional non-invasive rectal suppository dosage form, this approach offers several advantages. It reduces the incidence of adverse effects while enhancing drug bioavailability, opens up the possibility of small-scale laboratory and industrial production with simplified manufacturing processes, and accelerates the availability of the repositioned drug for patients with prolonged Lyme disease symptoms.

## Figures and Tables

**Table 1:** Determination of f_1_, f_2_, and Mahalanobis distance from the comparison of dissolution profiles for of the suppositories containing inclusion complexes or physical mixtures of DIS with HPBCD and RAMEB.

Compared Samples	TEST: phys. mix. (DIS + HPBCD) + PEG1500:PEG4000 50:50 Versus REFERENCE: incl. cplx. (DIS + HPBCD) + PEG1500:PEG4000 50:50	TEST: phys. mix. (DIS + RAMEB) + PEG1500:PEG4000 50:50 Versus REFERENCE: incl. cplx. (DIS + RAMEB) + PEG1500:PEG4000 50:50
f_1_ ± Standard Error	4.59 ± 1.09	12.48 ± 2.61
f_2_ ± Standard Error	80.88 ± 3.36	61.43 ± 4.78
Mahalanobis distance ^1^	2.11	1.16

^1^ Mahalanobis distance at the level for max mean difference of 15. Abbreviations: DIS = disulfiram, HPBCD = hydroxypropyl-β-cyclodextrin, incl.cplx. = inclusion complex, PEG = polyethylene glycol, phys.mix. = physical mixture, RAMEB = randomly methylated-β-cyclodextrin.

**Table 2:** Drug content variations over one-month stability.

Time	DIS Content (mg) [±SD]	DIS Content (%) [±SD]	Δ (Week_n_-Initial) (mg)	Δ (Week_n_-Initial) (%)
Initial	29 [±1.49]	96.66 [±4.98]	0	0
Week_1_	30.24 [±1.52]	100.79 [±5.07]	1.24	4.13
Week_2_	28.94 [±0.70]	96.46 [±2.32]	−0.06	−0.20
Week_3_	30.14 [±0.64]	100.48 [±2.14]	1.15	3.82
Week_4_	29.48 [±2.73]	98.26 [±9.11]	0.48	1.60

## Data Availability

The original contributions presented in the study are included in the article/Supplementary Materials, further inquiries can be directed to the corresponding authors.

## Supplementary Materials

The following supporting information can be downloaded at: https://www.mdpi.com/article/10.3390/pharmaceutics17070849/s1, Figure S1: The comparative Differential Scanning Calorimetry (DSC-A,B) and X-ray diffraction study (XRD-C,D) of of the free drug, raw CDs, physical mixtures and inclusion complexes; Figure S2: Particle size histogram and distribution fitting of disulfiram (DIS) (A), hydroxypropyl-β-cyclodextrin (HPBCD) (B), randomly methylated-β-cyclodextrin (RAMEB) (C), inclusion complex of DIS and HPBCD (D), inclusion complex of DIS and RAMEB (E); Figure S3: Dissolution profile comparison for formulation process optimization of the suppositories containing inclusion complexes or physical mixtures of DIS and HPBCD (A) or DIS and RAMEB (B). Dissolution profile comparison of the suppositories for CD type selection: HPBCD or RAMEB in physical mixture containing suppositories (C) and HPBCD or RAMEB in inclusion complexes containing samples (D); Figure S4: μFLUX (Pion Inc., Billerica MA, USA) apparatus setup used for determination of dissolution-permeation characteristics of the suppository samples with respective DIS concentrations on donor and acceptor sites; Figure S5: Characteristic peak of DIS at position 13.8 (°2θ [Cu]) in the DIS-containing samples.

## Abbreviations

The following abbreviations are used in this manuscript:

ALDH	Aldehyde Dehydrogenase
ATR-FTIR	Attenuated Total Reflectance Fourier-Transform Infrared Spectroscopy
CD	Cyclodextrin
DIS	Disulfiram
DS	Degrees of Substitution
DSC	Differential Scanning Calorimetry
f_1_	Difference Factor
f_2_	Similarity Factor
HF	Hard Fat
HPBCD	Hydroxypropyl-β-Cyclodextrin
incl.cplx.	Inclusion Complex
J(t)	Flux
P_eff_	Effective Permeability
PEG	Polyethylene Glycol
phys.mix.	Physical Mixture
PTLDS	Post-Treatment Lyme Disease Syndrome
RAMEB	Randomly Methylated-β-Cyclodextrin
XRD	X-ray Diffraction
