# Supplementary material for: Development of Low-Dose Disulfiram Rectal Suppository Intended for Application in Post-Treatment Lyme Disease Syndrome
Source: Pharmaceutics. 2025 Jun 28;17(7):849. doi: 10.3390/pharmaceutics17070849 (PMC12298725; doi:10.3390/pharmaceutics17070849)
Supplement: Supplementary file 1 [file pharmaceutics-17-00849-s001.zip › pharmaceutics-3683316-supplementary.pdf]

Supporting information

Screening Differential Scanning Calorimetry (DSC) and X-ray diffraction study (XRD) for inclusion complex formation

The physicochemical properties of the free drug molecule and the free cyclodextrin (CD) molecule are different from their counterparts in the complexed form. The changes in the physicochemical properties of drugs can be observed using several methods [1], such as DSC and XRD. DIS was characterized by a narrow endotherm with a minimum at 72°C, corresponding to the melting point and indicating its crystalline state. The starting CDs showed no sharp endothermic peaks, due to their amorphous structure, and only a broad endotherm between 50-140°C was observed, related to the evaporation of the absorbed water. In case of physical mixtures, the endotherm peaks with the minimum at 71°C (DIS+HPBCD) and 73°C (DIS+RAMEB)] appeared, corresponding to the melting point of the crystals of DIS. The inclusion complexes presented similar profiles to that of raw CDs, the melting peak of crystalline DIS was missing, attributed to the presence of molecular interactions between the guest and host molecules [2]. The XRD analysis of the pure drug displays a pattern with distinct series of accentuated peaks, indicating a crystalline structure, while HPBCD and RAMEB presented an amorphous pattern by a diffuse halo. The physical mixture contained a superimposition of strong peaks on a diffuse halo, representing the combination of crystalline (DIS) and amorphous (CDs) phases. Conversely, the patterns of the inclusion complexes lacked crystallinity-related intense peaks and showed a pattern like the raw CDs, providing evidence of amorphization [2].

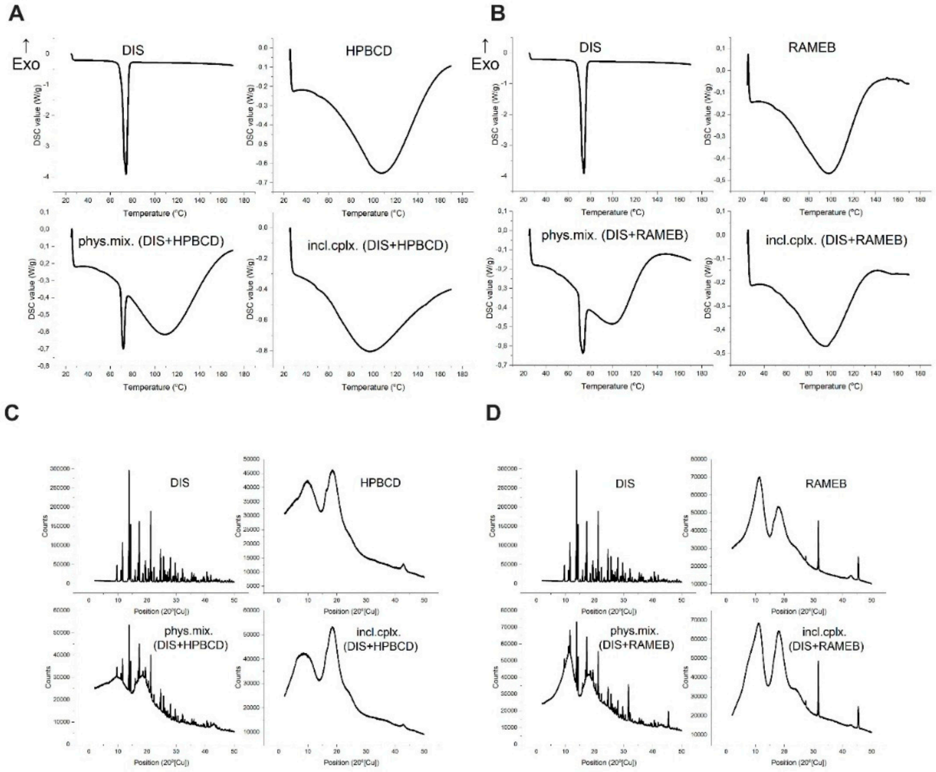

Figure S1. The comparative Differential Scanning Calorimetry (DSC-A,B) and X-ray diffraction study (XRD-C,D) of of the free drug, raw CDs, physical mixtures and inclusion complexes. Abbreviations: DIS=disulfiram, HPBCD= hydroxypropyl-β-cyclodextrin, incl.cplx.=inclusion complex, phys.mix.=physical mixture, RAMEB=randomly methylated-β-cyclodextrin.

Determination of average particle size (histogram) from scanning electron microscopy (SEM) image:

A JEOL JSM 6380LA type scanning electron microscope (JEOL, Tokyo, Japan) was applied to investigate the morphology of DIS and CDs. The SEM picture took was transferred into the ImageJ software and analyzed for particle size and size distribution, according to the method adopted from Mazzoli and Flavoni [3]. Firstly, a calibration was required in ImageJ to correlate the image dimensions in pixel to physical dimensions. The procedure consists in the drawing of a line over the scale bar of the image acquired by the SEM. For each substance, 100 particles were measured, and the measurement results were transferred to Origin 9.5 to create the histograms and apply particle size distribution fitting statistics. Lognormal fitting was used, as it gives the best fitting parameters in all the cases.

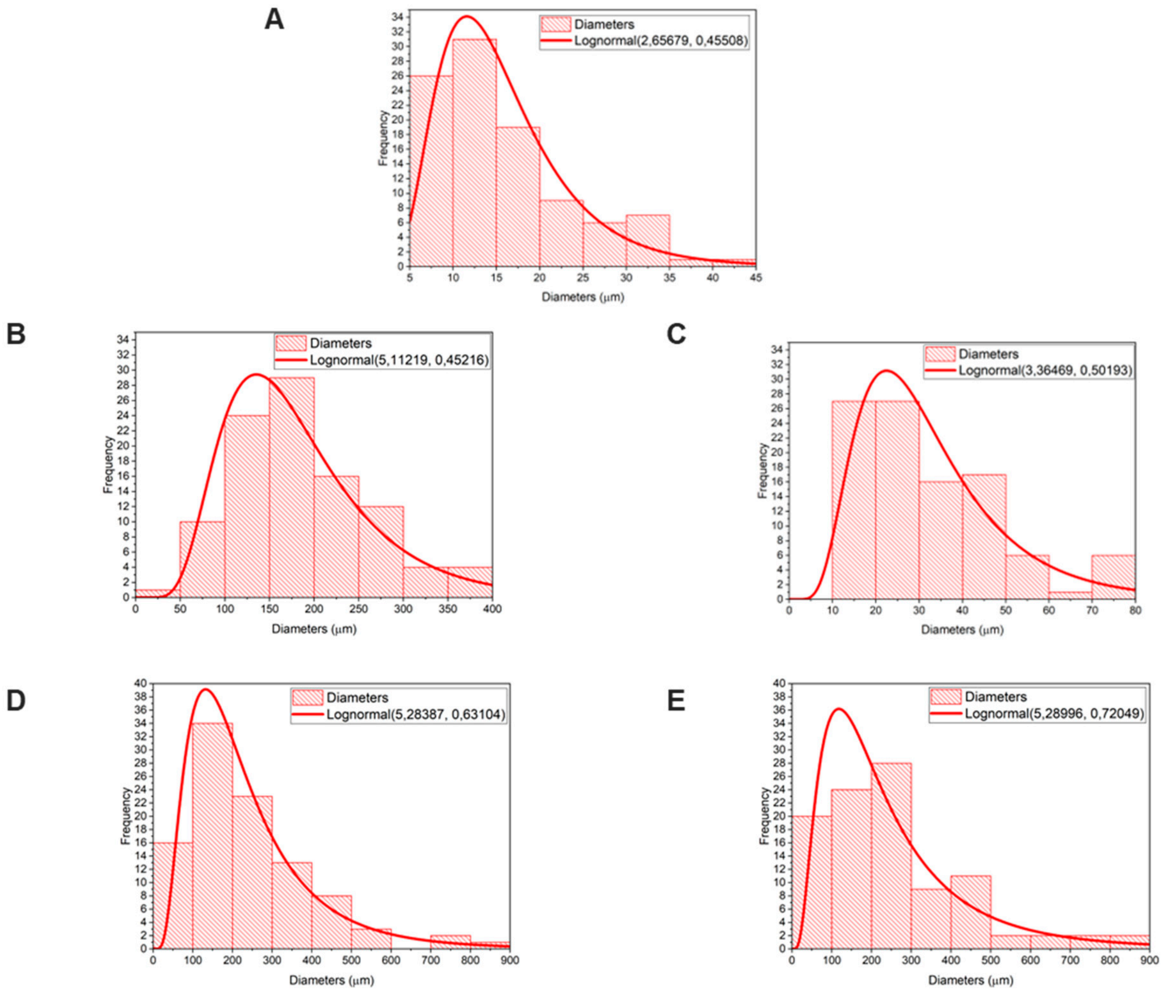

Figure S2. Particle size histogram and distribution fitting of disulfiram (DIS) (A), hydroxypropyl-β-cyclodextrin (HPBCD) (B), randomly methylated-β-cyclodextrin (RAMEB) (C), inclusion complex of DIS and HPBCD (D), inclusion complex of DIS and RAMEB (E)

Dissolution-driven justification of the preliminary inclusion complex preparation step in the suppository formulation process

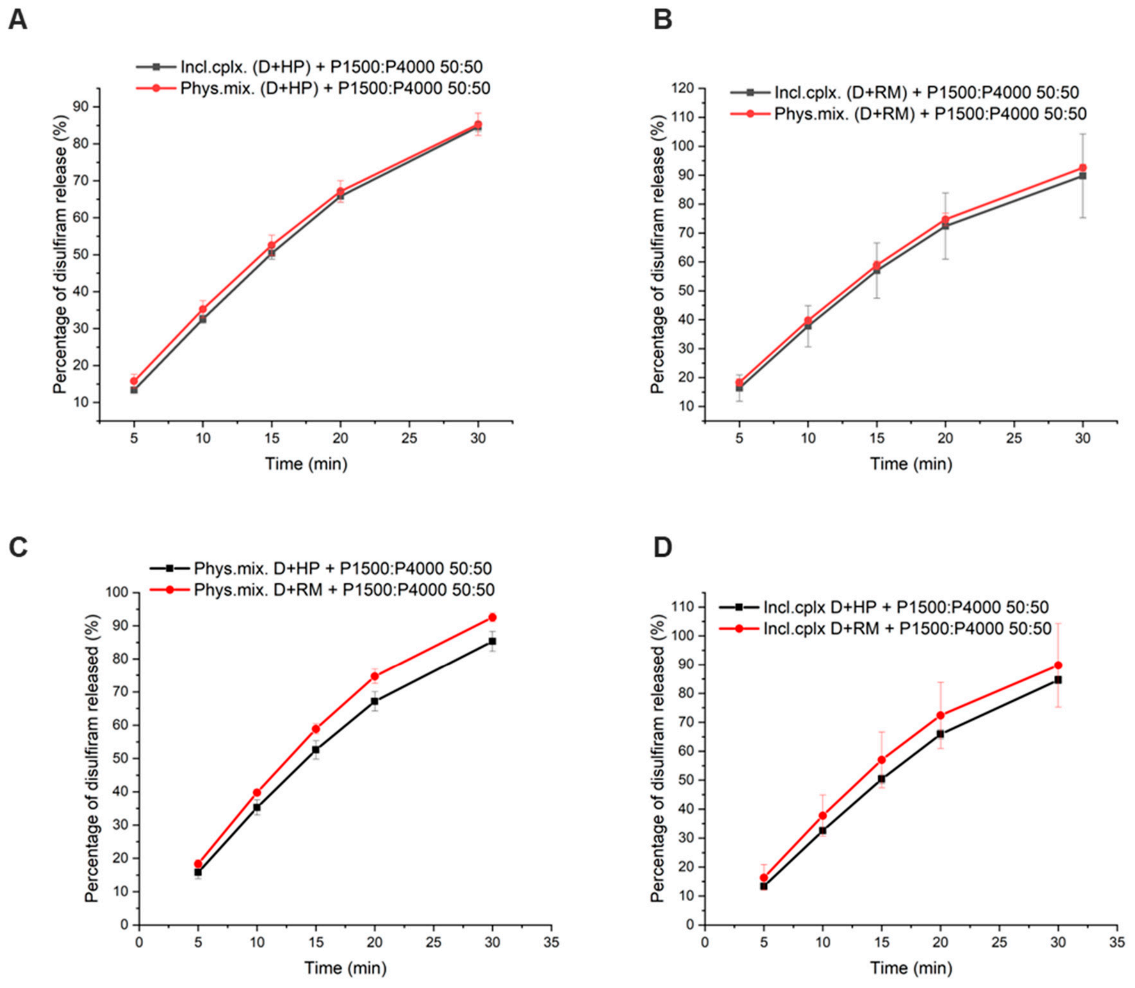

Figure S3. Dissolution profile comparison for formulation process optimization of the suppositories containing inclusion complexes or physical mixtures of DIS and HPBCD (A) or DIS and RAMEB (B). Dissolution profile comparison of the suppositories for CD type selection: HPBCD or RAMEB in physical mixture containing suppositories (C) and HPBCD or RAMEB in inclusion complexes containing samples (D). Abbreviations: DIS=disulfiram, HPBCD=hydroxypropyl-β-cyclodextrin, incl.cplx.=inclusion complex, P=polyethylene glycol, phys.mix.=physical mixture, RAMEB=randomly methylated-β-cyclodextrin, supp.=suppository.

Small volume dissolution-permeation analysis

The donor and the acceptor phase of the μFLUX (Pion Inc., Billerica MA, USA) apparatus were connected in a horizontal direction. The two horizontal chambers were divided by an impregnated synthetic membrane (polyvinylidene difluoride /PVDF/ membrane with 0.45 μm pores). The biomimetic membrane is placed into membrane holder (female and male parts). Tweezers are used to handle the membrane; and firstly, the dry membrane is placed into the female half of the membrane holder. 25 μL of n-dodecane lipid solution is pipetted directly onto the membrane and distributed across the entire membrane surface to ensure complete coverage. When the membrane is fully coated, it turns completely translucent. This step is followed by a time-sensitive procedure, within a few minutes (not more than 10 minutes) to prevent the membrane from drying out. The second O-ring is wetted with water and carefully pressed into the male half of the membrane holder, avoiding contact with the membrane. The two halves of the membrane holder are assembled, sandwiching the impregnated membrane between them. The crosshead magnetic stirrer bars are inserted into both chambers to ensure continuous stirring during the measurement time. The chambers are filled with the appropriate medium: donor chamber with pH=7.4 phosphate buffer and acceptor with Acceptor Sink Buffer (ASB). The volumes of the donor and the acceptor phase are the same (20.0 mL) with a 1.54 cm^2 diffusion area. The assembled and filled membrane holders are placed into the MicroFLUX apparatus. The UV-probes are immersed into the buffers, the existence of leaks and bubbles are monitored before the assay, then the experiment is started promptly. The chambers and UV-probes are wrapped with parafilm during the experiment to prevent leakage and evaporation.

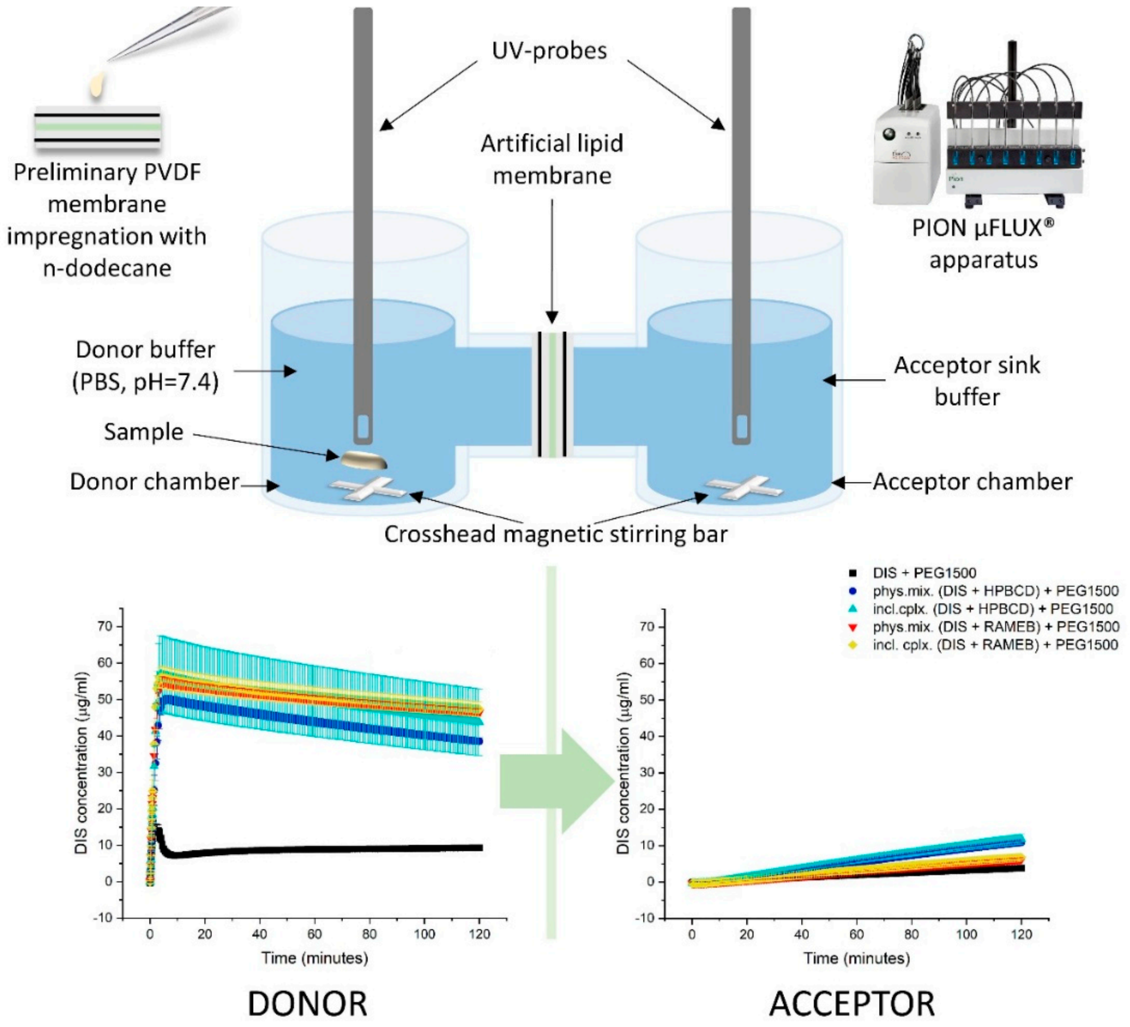

Figure S4. µFLUX (Pion Inc., Billerica MA, USA) apparatus setup used for determination of dissolution-permeation characteristics of the suppository samples with respective DIS concentrations on donor and acceptor sites. Abbreviations: DIS=disulfiram, HPBCD= hydroxypropyl-β-cyclodextrin, incl.cplx.=inclusion complex, PEG=polyethylene glycol, phys.mix.=physical mixture, RAMEB=randomly methylated-β-cyclodextrin.

XRD- Quality control of selected suppository composition

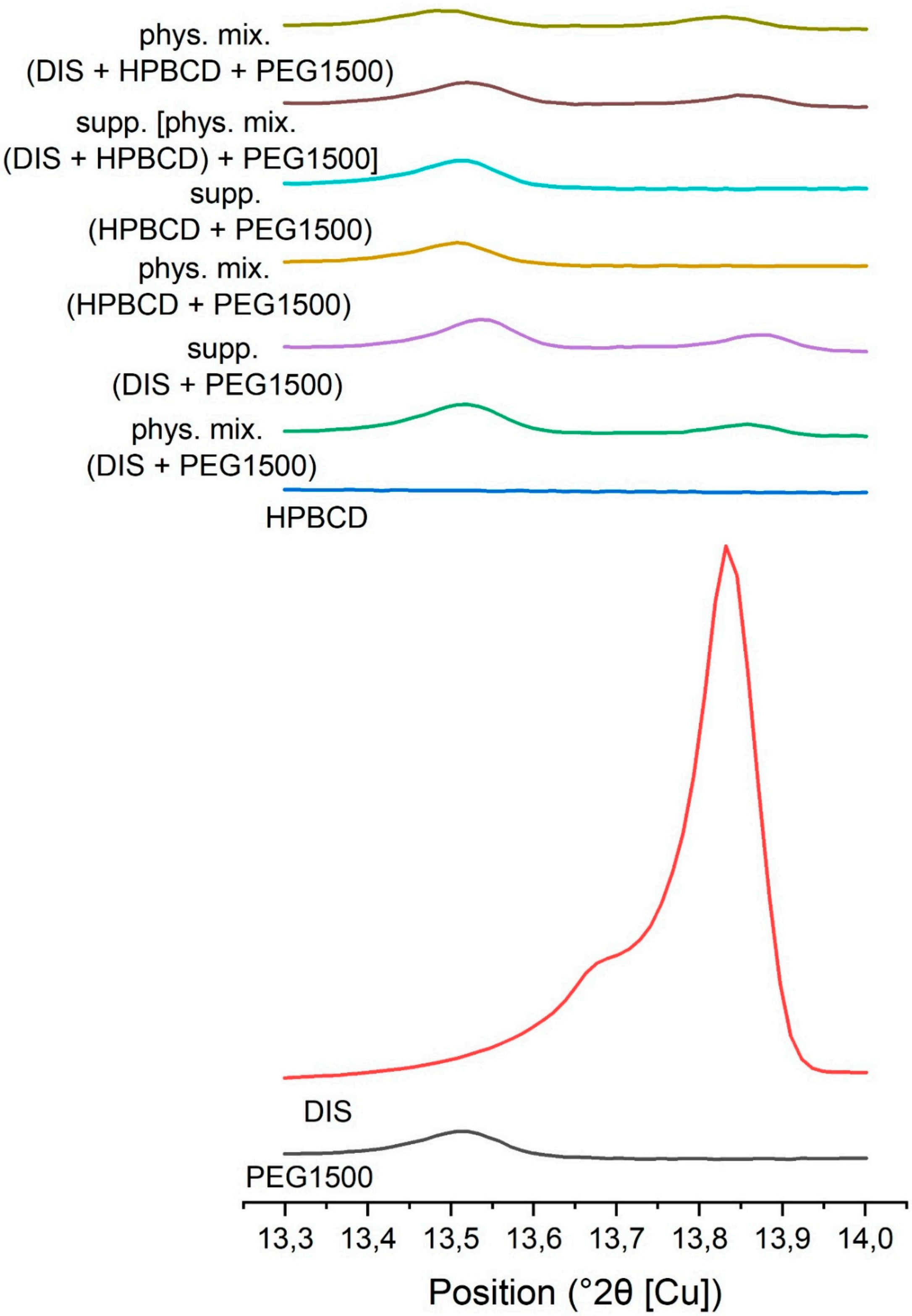

Figure S5. Characteristic peak of DIS at position 13.8 (°2θ [Cu]) in the DIS-containing samples. Abbreviations: DIS=disulfiram, HPBCD= hydroxypropyl-β-cyclodextrin, incl.cplx.=inclusion complex, PEG=polyethylene glycol, phys.mix.=physical mixture, RAMEB=randomly methylated-β-cyclodextrin, supp.=suppository.

References:

1. Jambhekar, S.S.; Breen, P. Cyclodextrins in pharmaceutical formulations I: structure and physicochemical properties, formation of complexes, and types of complex. *Drug Discov Today*. **2016,** *21(2),* 356-62. doi: 10.1016/j.drudis.2015.11.017
2. Benkő, B.M.; Tóth, G.; Moldvai, D.; Kádár, S.; Szabó, E.; Szabó, Z.I.; Kraszni, M.; Szente, L.; Fiser, B.; Sebestyén, A.; Zelkó, R.; Sebe, I. Cyclodextrin encapsulation enabling the anticancer repositioning of disulfiram: Preparation, analytical and in vitro biological characterization of the inclusion complexes. *Int J Pharm.* **2024**, *657*, 124187. doi: 10.1016/j.ijpharm.2024.124187.
3. Mazzoli, A.; Favoni, O. Particle size, size distribution and morphological evaluation of airborne dust particles of diverse woods by scanning electron microscopy and image processing program. *Powder Technol.* **2012**, *225*, 65–71.
